# The Relationship of Parent Support and Child Emotional Regulation to School Readiness

**DOI:** 10.3390/ijerph20064867

**Published:** 2023-03-10

**Authors:** Mei-Ling Lin, Richard A. Faldowski

**Affiliations:** 1 Department of Occupational Therapy, School of Health Professions, The University of Texas Health Science Center at San Antonio, San Antonio, TX 78229, USA; 2 Center for the Study of Aging and Human Development, Duke University Medical Center, Durham, NC 27708, USA; richard.faldowski@duke.edu

**Keywords:** emotional regulation, school readiness, parent supportiveness, Early Head Start Research and Evaluation Project

## Abstract

Using data from the longitudinal Early Head Start Research and Evaluation Project that were obtained when children were 14 through 60 months old, this study aims to explore the transactional effects between parent supportiveness and child emotion regulations skills. An autoregressive model with cross-lagged paths was utilized to examine the developmental trajectories of parent supportiveness and child emotion regulation, the directions of transactional relationships between them, and the transactional effects on the prediction of child cognitive school readiness. Significant autoregressive effects were found in both parent supportiveness and child emotion regulation trajectories. Significant concurrent and longitudinal transactional effects between these two processes were documented. The effects of child emotion regulation, parent supportiveness, and their transactional effects significantly predicted cognitive school readiness. This study exemplifies the use of archival longitudinal data to move beyond current unidirectional empirical understandings of child early psychosocial development toward more integrated perspectives. Equally important, the results provide critical insights for the timing of interventions as well as the involvement of parents in early intervention programs that early childhood educators and family services providers can benefit from.

## 1. Introduction

The transition to formal schooling is an ongoing process that is dually linked to the child’s future success in school life at one end and the child’s earlier home and preschool (if attended) experiences at the other end. Child school readiness, which is a construct that is used pervasively in educational research and school practice, is an indicator of the successful transition to formal schooling (i.e., kindergarten in the United States). This construct has been conceptualized as children’s developed capabilities—namely, their physical well-being and motor functions, social and emotional competencies, approaches toward learning, language abilities, and cognition and general knowledge—at approximately age five [1]. 

Physical and social stressors, such as unfavorable family dynamics, a disorganized household and neighborhood, irregular family routines, and inadequate emotion and learning supports from parents, which often are associated with financial insufficiency in children’s proximal environments, can impose detrimental effects on low-income children’s self-regulation and cognitive development [2,3]. These poverty-related stressors also can serve as malleable factors that can be addressed in early intervention programs for low-income children and families. For example, educating parents to provide positive regard, prompt but positive responses, and cognitive stimulation while interacting with their children is an integral and essential part of the nation-wide, federally funded Early Head Start (EHS) program. 

However, most prior studies have accounted for the reciprocal interactions between children and their contexts within a conceptual, but not statistical framework [1,4,5,6]. In other words, actual empirical evidence to support the complicated and potentially transactional (bidirectional) relationships between children and their proximal environments that are indicated in many child development theories is lacking. In order to address this deficiency in the current literature, this study empirically examined the age zero-to-three transactional effects between parental emotion and learning supports and child emotion regulation, and their relationships to child cognitive readiness at age five. 

## 2. Literature Review

### 2.1. Children’s Emotion Regulation Development: The Conceptual Framework

Two theoretical frameworks undergird this study: the family stress model and the bioecological model of human development. Together, these frameworks support this study’s investigation into the transactional relationships between parental supports and children’s early emotion regulation and help to address the urgent need to focus on low-income children. 

#### 2.1.1. Family Stress Model

According to the family stress model, compared to their economically more advantaged peers, children living and growing up in families with impoverished circumstances are at higher risk of experiencing self-regulation, language, and cognitive delays [3]. Owing to the limited financial resources available in the household and the increasing parental stress that results from financial distress, children living in low-income families are more likely to be exposed to less favorable family dynamics (e.g., more spousal conflict and turmoil, family dissolution, and domestic violence), disorganized or hazardous household and neighborhood environments [7], irregular family routines [8,9,10], and less emotional supports from parents, characterized by elevated parental harshness and diminished parent responsiveness [11]. Given the limited amount and quality of material goods and services that can be purchased by low-income families, these children are also more likely to have less cognitively stimulating home environments, characterized by, for example, fewer age-appropriate toys, fewer informal learning venues, fewer educational (digital) materials or available print media, and more passive exposure to television [12]. 

These poverty-related family characteristics can serve as chronic social and physical stressors in children’s immediate environments, thereby straining the development of biological response systems that maintain children’s equilibrium (e.g., self-regulation), language, and cognitive abilities. In order to inform early childhood intervention practices, more longitudinal outcomes research is needed to identify key malleable environmental factors and intervention timing for improving low-income children’s outcomes, which is an aim of this study. 

#### 2.1.2. Bioecological Model of Human Development

The direction of the impacts between low-income children and their surrounding environments is not one-way but potentially two-way or transactional. Bronfenbrenner’s bioecological model of human development suggests that optimal child development occurs through children’s regular and ongoing participation in ‘proximal processes’ that constitute progressively more complicated reciprocal interactions between an active, evolving child and the people, objects, and symbols in his/her immediate environments [13]. Bronfenbrenner’s bioecological model provides insights for developmentalists to embed the reciprocal relationships between young children and parenting practices in their statistical models. By incorporating the bioecological model, this study thus contributes to a more integrated understanding of children’s early developmental outcomes by revealing both the longitudinal effects of parental supports on child emotional regulation and vice versa. 

### 2.2. Children’s Emotion Regulation Development: Perspectives from Empirical Studies

In this section, I first review the definition and significance of ‘emotion regulation’ in order to justify its selection as an outcome variable in this study. Then, I synthesize empirical evidence from child development and family studies as well as from social policy and early intervention research to specify current empirical understandings regarding the effects of supportive parent–child interactions on children’s early developmental trajectories of emotion regulation in low-income families.

#### 2.2.1. Child Emotion Regulation: Definition and Significance

‘Emotion regulation’ refers to a complex and interrelated process of “initiating, avoiding, inhibiting, maintaining, or modulating the occurrence, form, intensity, or duration of internal feeling states, emotion-related physiological and attentional processes, motivational states, and/or behavioral concomitants of emotion” that allow children to interpret, translate, and respond to social cues appropriately in their living environments [14]. A child’s emotion regulation is crucial for organizing his/her behaviors and further contributes to psychosocial competencies (e.g., self-regulation, interactive and pro-social behaviors, and communication) in toddlerhood [15]. Additionally, emotion regulation and cognitive development are closely aligned [16]. Between the ages of zero to three, both the baseline levels and the growth rates of cognition have been found to be significantly related to the baseline levels and changes in emotion (task-oriented) regulation competencies [17]. 

Children who are able to better manage their emotions and behaviors during challenging tasks and in challenging settings are better able to attend to tasks [18,19], have greater motivation and exploration for learning [20], and make better use of their learning environments [21] at kindergarten entry. From the points of view of 3000 kindergarten teachers, a child’s ability to verbalize needs and thoughts, follow directions, take turns, not disrupt classroom activities, and be sensitive to others—all considered indicators of socio-emotional competence—are characteristics that make children ready to learn in school [15]. 

#### 2.2.2. Child Emotion Regulation: The Role of Supportive Parenting

Importantly, children’s self-regulatory abilities for managing their emotions and behaviors are established within nurturing family processes and practices, specifically through actual involvement in parental emotion socialization practices that are characterized by parents’ overt and covert behaviors (e.g., parental reactions to children’s emotional expressions and discussion of emotion) in response to children’s emotional expression and regulation [22,23]. Mediational analyses of a sample of 119 children, aged 12 to 36 months, showed that a mother’s contingent responsive behaviors mediated the relationship between maternal emotion socialization practices and toddlers’ socio-emotional competence (i.e., compliance, empathy, mastery and motivation, imitation, and play) [23]. Furthermore, among fourth and fifth graders from diverse ethnic backgrounds, the linkage between positive family climate, parental warmth and sensitivity and children’s emotion regulation remained significant at these later ages [24]. Such findings have inspired additional investigations into the effects of parent supportive behaviors during parent–child interactions on child emotion regulation outcomes. 

Parental emotion supports, characterized by sensitivity, engaged attention, emotional flexibility, positive regard, and encouragement of autonomy, thus might reflect a subtle form of emotion socialization practices [25] through which children can learn how to manage their emotions [4]. Certain parent–child interaction findings show that interaction quality, indicated by the presence and adequacy of parental emotion supports, can significantly mediate the relationship between parent–child interaction and child self-regulation during children’s first three years [25]. Overall, ongoing and consistent emotional supports from parents provide a nurturing environment in which children’s cues, interests, and explorations are encouraged, thus enhancing young children’s motivation to learn [26].

#### 2.2.3. Linking Parent Supportiveness to Low-Income Children’s Outcomes

Along with the availability of cognitively-stimulating resources and activity opportunities, the early provision of emotional supports from parents is associated with low-income children’s early self-regulation and later cognitive school readiness [27,28,29,30]. Parent supportiveness (conceptualized somewhat similarly to parents’ emotional supports), which includes parental sensitivity, cognitive stimulation to learning, and positive regard, has served as a predictor of child outcomes in many studies that have used samples of low-income families that participated in the Early Head Start Research and Evaluation Project (EHSREP). 

Focusing on low-income African American families, Bocknek, Brophy-Herb and Banerjee [4] found that the rates of change in maternal supportiveness predicted the rates of change in child emotion regulation, as measured when children were approximately 14, 24, and 36 months old. Extending the inquiry to other ethnically diverse low-income families, Brophy-Herb, Zajicek-Farber, Bocknek, McKelvey and Stansbury [31] found that both the initial status (intercept) and rate of change (slope) in maternal supportiveness predicted toddlers’ emotion regulation at 14 months and its change over time. Moreover, children whose mothers displayed high stable supports when their children were zero to three years of age scored best in tests of vocabulary and appropriate behaviors at age five [32]. Additionally, Chazan-Cohen, Raikes, Brooks-Gunn, Ayoub, Pan, Kisker, Roggman and Fuligni [29] found that sustaining the provision of parental supports across the child’s first five years predicted children’s vocabulary (letter–word knowledge) and emotion regulation abilities at approximately age five. 

Conversely, other studies focused on the negative effects of fewer parental emotional and learning supports on children’s early outcomes. Children growing up in chronic poverty are at risk of having ‘psychologically absent’ parents who are physically present yet psychologically and emotionally absent in family roles. Findings from a sample of 2632 mother–child dyads indicate that psychological and emotional absence measured at children’s 36-month assessment significantly predicted their concurrent task orientation and emotion regulation [33]. Additionally, Lugo-Gil and Tamis-LeMonda [34] found that less sensitive parenting was significantly associated with children’s negative cognitive development in early childhood. Fuligni, Brady-Smith, Tamis-LeMonda, Bradley, Chazan-Cohen, Boyce and Brooks-Gunn [32] showed that observed lower levels of supports that children experienced at any of the three age points (i.e., 14, 24, and 36 months) in their study predicted lower language scores at age five. It can be concluded that experiencing fewer parental supports in early childhood can adversely impact not only children’s early emotion regulation outcomes, but also their language and cognitive outcomes at kindergarten entry.

### 2.3. The Current Study

Research studies that have used samples of low-income families that participated in the EHSREP provide evidence that mothers’ ongoing and stable provision of emotional and learning supports to infants and toddlers can mitigate the detrimental effects of poverty and related familial risks (e.g., single parenthood, unemployment, receipt of monetary public assistance, and severe maternal depression) on children’s early emotion regulation development and cognitive school readiness outcomes [27,28,29,30]. Yet, most of these studies reported only the positive effects of parent supports or healthy family routines on low-income children’s health and development, not the reverse or bidirectional relationships [4,29,30]. This study fills this gap in the literature by investigating the transactional effects of parental supports and early child emotion regulation and their relationships to cognitive school readiness outcomes. The specific research questions are: 

(1) How do intra-individual developmental trajectories of parent supportiveness and child emotion regulation change over time? 

(2) What are the directions of transactional effects between parent supportiveness and child emotion regulation? 

(3) How well do the transactional effects of parent supportiveness and child emotion regulation predict age five cognitive school readiness?

(4) How do EHS program status and race/ethnicity predict the child and parent outcomes? 

## 3. Method

### 3.1. Study Design

This study includes secondary analysis of longitudinal data obtained from the EHSREP; the database consists of a sample of 3001 children and their primary caregivers who were income eligible for the EHS program at 17 selected sites around the country. The characteristics of the 17 sites reflected all EHS programs that were in operation during 1996 and 1997 [35,36]. Participating families were randomly assigned to receive either the EHS program plus local community services (n = 1503, program group) or usual community-based children and family services alone (n = 1474, control group) from pregnancy through to the child’s third birthday [35,36]. Comparative analysis results revealed that the control families shared similar demographic characteristics with families that were served by EHS [36]. Trained (bilingual) interviewers and assessors followed strict protocols and met rigorous criteria for reliability [35]. All participating families completed informed consent forms that followed ethical conduct for research using human subjects. The current study also was approved by the University of North Carolina at Chapel Hill Institutional Review Board.

EHS is a federally funded, two-generation early intervention program that targets primarily low-income children and families. EHS begins services during pregnancy and continues until the child’s third birthday. It seeks to promote child development by providing comprehensive parenting education, family supports for self-efficacy and healthy functioning, and direct child services. Over 1000 EHS programs have served over 145,000 families since the first 68 EHS programs were funded in 1995. EHS may serve families through home-based services (i.e., weekly 90 min home visits and group socialization meetings for parents twice a month), center-based services (i.e., high-quality center care combined with a minimum of two home visits per year), or a combination of these two service options. Each type of program also provides comprehensive family supports and referrals to related community agencies that address specific needs of families. 

The EHSREP documented the outcomes of children who received either EHS or other community programs through 5th grade. Children and family data were collected through in-home direct child assessments, parent interviews, and videotaped observations of parent–child interactions when the children were approximately 14, 24, 36, and 60 months old and in 5th grade. This study used the child assessments and coder ratings for parent–child interaction quality (i.e., parent supportiveness) from the first four data collection waves. Child assessment booklets and parent–child interaction videotaped protocols can be downloaded from the ACF website: https://www.acf.hhs.gov/opre/research/project/early-head-start-research-and-evaluation-project-ehsre-1996-2010 (accessed on 15 June 2018).

Because of the data availability on child, parent, and family levels and across the early childhood time points, the dataset from the longitudinal Early Head Start Research and Evaluation Project was selected to answer the proposed research questions. 

### 3.2. Participants

EHSREP participants in the program and control groups were combined for analyses, given the project’s focus on exploring the potential transactional relationships of parent supportiveness and child emotional regulation rather than a focus on between-group differences. The current study included only those parent–child dyads that had at least one wave of data present at the 14-, 24-, or 36-month assessments, which yielded a final longitudinal sample of 2132 parent–child dyads. Data from families without any data for key study measurements (No Data) or for whom data were obtained outside the assessment window set for each wave of birthday-related child assessments and video-taped parent–child interactions (Outside Window) were excluded. Sample sizes were varied across measures and time points (See Table 1).

Extensive attrition analyses were conducted within the samples to examine patterns of missingness. No significant differences were evident between groups based on missingness or that were related to key predictor or outcome variables, and data were determined to be missing at random. Missing data were inputted prior to analysis using the full information maximum likelihood (FIML) estimation algorithm in Mplus 7.4 software [37]. FIML estimation is an efficient method of dealing with substantial portions of missing data under missing-at-random assumptions by estimating the models using all available information from all cases [38]. 

Family Demographics. The biological mothers of the focus children were the vast majority of the study respondents in the original dataset (n = 2960, 99%). The mean maternal age at EHSREP enrollment was 22.89 years (*SD* = 5.88 years). Within the current sample, the mean ages of toddlers at their age-related assessments was 14.84 months (*SD* = 1.2 months), 25.15 months (*SD* = 1.52 months), and 37.15 months (*SD* = 1.51 months), whereas the mean age at the children’s final assessment before entering kindergarten was 63.14 months (*SD* = 4.02 months). Table 1 presents additional family demographics. 

### 3.3. Measures

**Figure 1:**
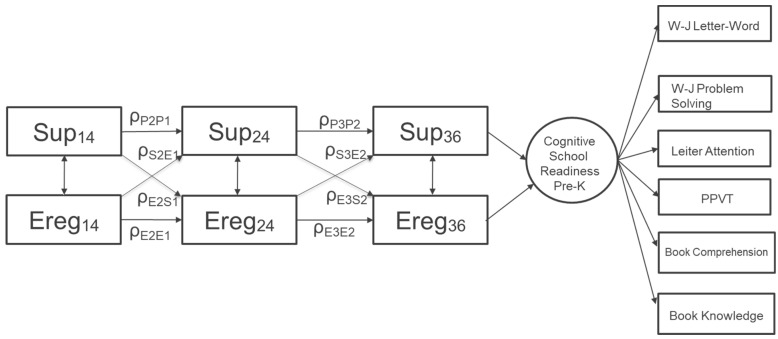
A hypothesized generalized autoregressive model for two series of repeated measures, including cross-lagged effects between predictors and a distal outcome.

Child Emotion Regulation. For this study, children’s competence in emotion regulation was based on their ability to change tasks and test materials, display limited negative affect, and manage frustration with tasks during the assessment. At each of the assessment points (i.e., 14, 24, and 36 months), emotion regulation was computed as the mean of the examiner’s ratings for seven items from the standardized Bayley Scales of Infant Development (2nd Edition)—Behavioral Rating Scales (BSID-II) [39]. Each item was rated on a 5-point scale, ranging from not at all (1) to all the time (5), with higher scores indicating more positive responses. This scale has been used extensively as an indicator of children’s self-regulatory competence [30]. In Figure 1, I use ‘E’ to indicate the child emotion regulation variable.

Parent Supportiveness. Parent supportiveness was measured at the three waves (i.e., ages 14, 24, and 36 months) through videotaped parent–child interactions during semi-structured play using the three-bag task [40]. During the ten-minute task that was intended to elicit naturally occurring behaviors of parents and children, mothers were instructed to sequentially open three bags that each contained an age-appropriate book and toys. Additionally, mothers had the flexibility to determine whether and when to transition between the different bags as well as the degree of her directiveness in the play. Both parent and child behaviors were coded from the videotapes. Each dimension of parenting behavior was rated on a 7-point scale, ranging from very low (1) to very high (7). The parent supportiveness score was a mean of three highly correlated positive parenting behaviors (*r* = 0.67 to 0.96, *p* < 0.001): sensitivity, stimulation of cognitive development, and positive regard. 

Cognitive School Readiness. Cognitive school readiness was estimated at the 60-month assessment by using six standardized (*M* = 100, *SD* = 15) measures, including the Letter–Word Identification and the Applied Problem Solving subtests in the Woodcock-Johnson III tests of cognitive academic competence [41], Receptive Vocabulary subtest in the Peabody Picture Vocabulary Test (3rd edition) (PPVT-III) [42], the Attention subtest in the Leiter International Performance Scale (3rd edition) (Leiter-3) [43], and Book Comprehension and Book Knowledge sections in the Story and Print Concepts measure. All tests have been well validated and normed for various ages and genders. In addition, both subtests in Woodcock-Johnson III have been translated into Spanish and have been used by researchers to assess the academic competence of children at kindergarten entry [44].

### 3.4. Statistical Analysis 

The sets of parent supportiveness and child emotion regulation variables over the three time points and the cognitive school readiness variable were utilized in a generalized autoregressive model to examine intra-individual developmental trajectories of children’s and their parents’ outcomes as well as the direction of the transactions between them. The analytic strategy is based on Bollen and Curran’s (2004) recommendations [45]. First, univariate unconditional autoregressive models were estimated for one developmental process (parent supportiveness or child emotion regulation) at a time. These estimations are not reported here because these models yielded conclusions that were identical to those obtained from the multivariate models. Second, the cross-lagged regression parameters from child emotion regulation to parent supportiveness and vice versa were estimated. Finally, the covariates of program status and race, and the distal outcome of cognitive school readiness were added into the model to determine whether this model is still a complete and discrete representation of the sample. 

We imposed equality constraints to achieve model identification [46]. Accordingly, we imposed two types of equality constraints on longitudinal paths that involve the same sequence of variables (i.e., parent supportiveness and child emotion regulation), resulting in within-variable autoregressive paths and cross-lagged paths in the model that held constant from the first to the third time points. The fit of all the models was evaluated using multiple indices: the X^2^ likelihood ratio test, the comparative fit index (CFI), the Tucker–Lewis index (TLI), the standardized root mean square residual (SRMR), and the root mean square error of approximation (RMESA). Values greater than 0.90 for the CFI and TLI are indicative of adequate fit, although values greater than 0.95 are preferable [47,48]. Values smaller than 0.06 for the RMESA and smaller than 0.08 for the SRMR are indicative of good fit [47,48]. 

## 4. Findings

### 4.1. Descriptive Statistics

Table 2 presents the descriptive statistics and correlations for all the study variables. These results show that the various measurement points for parent supportiveness and child emotion regulation are moderately correlated to one another. In general, the level of relatedness is strongest between adjacent time points.

### 4.2. Generalized Autoregressive Models

**Figure 2:**
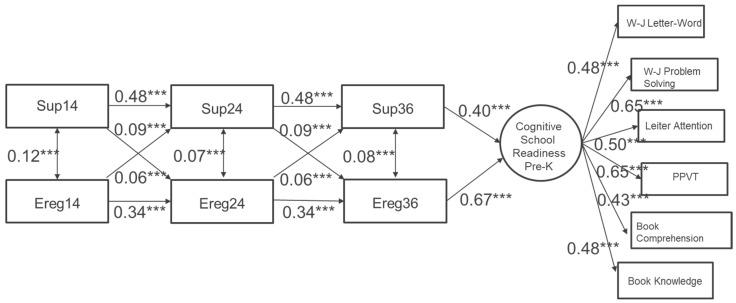
A final generalized autoregressive model for repeated measures of parent supportiveness and child emotion regulation with cross-lagged effects and a distal outcome. *** *p* ≤ 0.001.

The results reveal in Figure 2 that the generalized autoregressive model provides an acceptable fit to the data (*x*^2^ (52) = 302.32, *p* = 0.00; CFI = 0.94; TLI = 0.91; RMESA = 0.05; SRMR = 0.06). Regarding Research Question 1: The developmental trajectories of the two developmental processes (i.e., parent supportiveness and child emotion regulation), the autoregressive paths for each process can be constrained to equality. An interpretation of the autoregression coefficients is the level of consistency over the period of study/observation time. The estimated autoregression coefficients indicate a moderate tendency to remain consistently high or low for parent supportiveness and a modest tendency to remain consistent for child emotion regulation.

In terms of Research Question 2: The transactional effects between the two phenomena, the results show that both cross-lagged regressions between parent supportiveness and child emotion regulation can be constrained to equality and appear to be statistically significant. Additionally, the time-specific correlations between parent supportiveness and child emotion regulation, though small, all appeared to be statistically significant. 

Regarding Research Question 3, the latent construct of cognitive school readiness was measured equally well at kindergarten entry by the six direct child assessments. Cognitive school readiness was significantly predicted by children’s 36-month emotion regulation capabilities and the parental emotion and learning supports that were provided at that same time point. As such, these findings automatically consider the evidence of autoregressive and transactional effects for both and between the two developmental processes during children’s first three years.

Lastly, to address Research Question 4, Table 3 presents the results from the regression of the model parameters on the predictors and their interactions. Family race predicted the levels of emotion and learning supports mothers provided to their children during play assessment. As children grew up, the racial effects on maternal supports faded out. Racial difference was also found in children’s cognitive school readiness outcome. Whether in EHS or comparison group significantly predicted the levels of maternal supports to their children, except when children were about 24 months old. 

## 5. Discussion

### 5.1. How Does the Intra-Individual Developmental Trajectory of Parent Supportiveness Change over Time?

The findings clearly show that later parent supportiveness and child emotion regulation are significantly predicted by their earlier measures. A moderate tendency to remain consistently high or low is evident for the parental emotion and learning supports provided to children during play. This finding is similar to the previous in-depth investigations into parent supportiveness trajectories using the EHSREP dataset. Using a sample of 1095 parent–child dyads from EHS group, Fuligni, Brady-Smith, Tamis-LeMonda, Bradley, Chazan-Cohen, Boyce and Brooks-Gunn [32] discovered four growth patterns, i.e., high stable, low stable, increasing, and decreasing, across European, African, and Latino groups. Additionally, these growth patterns of maternal supports were not associated with child gender or teen mother status for any of the ethnic groups [32]. Uniquely, the present study utilized the sample of parent–child dyads from both the EHS and comparison groups. The significant autoregressive paths identified between adjacent time points provide additional evidence for how well the earlier levels of parental supports can have an impact on later observations. Thus, this study contributes to the current literature regarding changes in earlier provision of parental supports in economically disadvantaged families. 

### 5.2. How Do EHS Program Status and Race/Ethnicity Predict the Parent Supportiveness Outcome?

Moreover, by including the ethnicity and program status as predictors of parent supportiveness in the autoregressive model, this study found the ethnic differences on the trajectory of parent supportiveness (except for the 36-month outcome) and child cognitive school readiness outcome. Similarly, Fuligni and Brooks-Gunn [40] revealed ethnic differences in mean levels of supportiveness across four growth patterns. Additionally, these ethnic differences in supportive parent–child interactions decreased as children aged. In contrast, Iruka’s (2009) study showed that during the 36-month assessment, European American mothers were observed to be more sensitive and less negative, and to provide more cognitively facilitating opportunities while interacting with their children compared to African American and Hispanic American mothers [49]. One possible reason for this contradictory finding is the selection of variables. During the 36-month assessment, in addition to parent supportiveness variable, there were *supportive presence* and *quality of assistance* variables available for researchers to create an overall composite variable for sensitive parenting. Since these two variables are not available in 14- and 24-month waves to allow for longitudinal investigation, the present study only used the parent supportiveness variable.

Additionally, this study found the positive program effects for parenting supports during 14- and 36-month assessments. Comparably, impact studies have found positive EHS influences on parenting and home environment outcomes when children were aged two and three. Mothers in the program group exhibited more supportiveness during play than their counterparts (*ES* = 0.09, *p* < 0.10 and *ES* = 0.15, *p* < 0.01) [50]. Program status was treated as a time-invariant in the current analysis, which in fact may be treated as a time-variant, leading to the difference between each finding. Future studies should take extra caution while interpreting the EHS program effects on parent, children, or family outcomes. 

### 5.3. How Does the Intra-Individual Developmental Trajectory of Child Emotion Regulation Change over Time?

Regarding the developmental trajectory of child emotion regulation, the autoregression paths revealed a modest tendency for child emotion regulation abilities to remain consistently high or low. In other words, children’s abilities to change tasks and test materials, limit displays of negative affect, and manage task frustration during the assessment remained less stable during the period from the ages of zero through to three in comparison to parent supportiveness but had a tendency to increase over time. This finding is not surprising given that children are developing rapidly during this period of time. Nonetheless, Raikes and colleagues’ study of a sample of 2441 low-income children also found an increase in children’s self-regulation abilities [30]. Moreover, the individual variations were most noticeable in both the rate of growth (slope) and at the 36-month time point [30]. As with the developmental trajectory of parent supportiveness, this study contributes unique information about how well children’s earlier developed emotion regulation capabilities can predict their later capabilities in this same domain.

### 5.4. How Well Do the Transactional Effects of Parent Supportiveness and Child Emotion Regulation Predict Age Five Cognitive School Readiness?

Further, this study sought to explore the directions of transactional relationships between parent supportiveness and child emotion regulation. The transactional effects between child and parent outcomes can translate to the significant longitudinal cross-lagged effects as well as the significant concurrent correlations between parent supportiveness and child emotion regulation. In other words, parent supportiveness and child emotion regulation mutually impact each other, both concurrently and longitudinally. Essentially, after considering the autoregressive effects of two developmental processes, the magnitude of the transactional effects appeared small (0.06 to 0.12). This finding suggests that factors other than parent supportiveness may also be predictive of child emotion regulation. Raikes and colleagues found that children who showed high degrees of anger, hostility, or negativity toward their parent(s) at 14, 24 and 36 months old developed their self-regulatory abilities at slower rates compared to their counterparts. Thus, future research should consider environmental and developmental factors (e.g., prenatal depression, poor maternal–child bonding) for children who are temperamentally difficult at such an early age and further explore the parental supportiveness variable as a predictor of later child outcomes. 

The significant effects detected regarding the longitudinal transactional effects of children’s emotion regulation and parent supportiveness contribute to a more nuanced understanding of children’s early emotion regulation development by moving beyond the current unidirectional understanding of the positive effects of supportive parenting interactions on child outcomes. For comparison, Luebbe, Kiel and Buss [51] study of children’s behavioral and/or emotional problems and parents’ responses to emotions (RTEs) found a reciprocal relationship between children’s internalizing behavior (i.e., more withdrawn, sad, or fearful) and mothers’ RTEs. That is, a two-year-old child’s internalizing behavioral problems could predict an increase in the mother’s use of supportive strategies (i.e., encouragement of emotion expression and problem solving), and a mother’s use of unsupportive strategies (i.e., punishing expressions, minimizing children’s emotion responses) could predict an increase in her two-year-old child’s internalizing problems across one year. 

Such findings suggest that future researchers should examine children’s internalizing or externalizing problems as moderators of the effects of parent supportiveness on child outcomes, which may in turn affect the likelihood of finding transactional relationships between child emotion regulation and parent supportiveness. This study is an initial attempt to investigate the transactional relationships between parent and child outcomes in the context of poverty. Although the bidirectionality or reciprocity of influences within proximal processes has been articulated in Bronfenbrenner’s bioecological model of human development, studying the effects of children’s emotion regulation abilities on parent supports for typically developing children still needs further empirical evidence. 

Moreover, the present study aimed to understand how well the transactional effects of parent supportiveness and child emotion regulation predict age five cognitive school readiness. The findings show that the cognitive school readiness construct was significantly predicted by both child emotion regulation and parent supportiveness at 36 months as well as their autoregressive and transactional effects. Consistently, Brophy-Herb, Zajicek-Farber, Bocknek, McKelvey and Stansbury [31] revealed the identical findings using latent growth curve analysis. Differently, the cognitive school readiness construct in the present study was estimated by six rather than two standardized assessments which measure children’s academic competence, receptive vocabulary, readiness to learn, and literacy at kindergarten entry. The significant findings from the extensive inclusion and validation of measures in the CFA model of cognitive school readiness provide important nuances to this construct. The implication for future research is that, when examining child outcomes, both child and parenting factors and their interactions need to be taken into consideration.

### 5.5. How Do EHS Program Status and Race/Ethnicity Predict the Child Cognitive School Readiness? 

Lastly, being in a certain racial group predicts children’s cognitive school readiness outcome. European American children had a higher possibility to perform well than African and Hispanic American children on the early achievement and vocabulary test scores at age 5 (i.e., Woodcock Johnson Letter–Word, Applied Problems, Leiter Attention Sustained, PPVT, and Story and Print Concept). Future research could further explore whether this finding is site specific because each EHS research site has a different racial composition and unique characteristics (e.g., teenaged mom is the majority). 

### 5.6. Implications for Early Childhood Practices

The reciprocity of parent supportiveness and child emotion regulation constitutes almost all kinds of things and activities parents do with their young children, which is the central focus in early childhood prevention, promotion, and intervention practice and research. This study provides empirical evidence to support early childhood practitioners to use a family-centered approach to work with children with socioemotional difficulties and their parents. When working with vulnerable populations (e.g., low-income families), building up an effective therapeutic relationship might be the most important step toward involving parents in the intervention process and enabling parents to make adjustments in their parenting practices to fulfill the goals they set for their children, for themselves, and for the whole family. With this component-level evidence in hands, early childhood practitioners are strongly encouraged to incorporate routine-based approach in their practice to walk parents through the child and family activities and routines impacted by children’s emotion regulation difficulties, and to enhance mutual performance and engagement in those important activities by coaching parents to provide emotion and learning supports to their children.

Based on the findings from the present study, the implication for future EHS program and other early intervention programs targeting at-risk families can be summarized as follows: EHS intervention should begin as early as about one year old; either taking a center-based, home-based, or combination intervention approach, a sufficient amount of time should be directly spent on issues or concerns related to parenting skills, children’s developing skills, and the parent–child interactions.

### 5.7. Study Limitations

Even with a large sample size, this study had some limitations. Most notably, the study was unable to address participant selection issues because of the secondary data analysis framework and reliance on extant data that limited the types of research questions that could be asked. Additionally, earlier transactional effects between parent supportiveness and child emotion regulation cannot be verified because the EHSREP data collection started when the children were aged 14 months. Obviously, child–environment transactions are meant to be more than just longitudinal reciprocal and concurrent correlational relationships between child emotional regulation performance and parental emotional and learning supports. Future research should be deliberately designed to target other parenting and child outcome variables and/or select different analytic methods to address their potential transactional relationships. 

## 6. Conclusions

Maximizing the benefit of an old yet important longitudinal dataset, this study moves beyond the unidirectional understandings of children’s early psychosocial development documented in previous empirical studies towards a more holistic and integrated perspective. The findings from the statistical model clearly showed that during the period from birth to three years old, both later parent supportiveness and child emotion regulation were significantly predicted by their earlier measures. Further, there were detectable concurrent and longitudinal transactional effects between parent and children outcomes. Ultimately, the cognitive school readiness construct was predicted by both child emotion regulation and parent supportiveness at 36 months, as well as their autoregressive and transactional effects. In contrast to the sole inclusion of mothers in existing OS studies of family routines, this study demonstrates the importance of focusing on both children and their parents, as well as considering the parent–child interaction and the temporal aspect to this relationship in studies of families with young children. 

## Figures and Tables

**Table 1:** Additional Family Demographics.

	N (%) or Mean (SD)
Family Race	
White	1086 (37%)
African American	1014 (34%)
Hispanic	692 (23%)
Other ^1^	133 (5%)
Number of Family Risks ^2^	
0–2	1129 (38%)
3	835 (28%)
4–5	704 (24%)
Primary Caregiver Role	
Mother	2960 (99%)
Parent Educational Attainment	
<12 years of schooling	1367 (45.9%)
12 years of school or GED	822(27.6%)
>12 years of schooling	681(22.9%)
Child Gender	
Male	1502 (51%)
Female	1446 (49%)
Child Age (in months)	
14 M	15 (1.2)
24 M	25 (1.3)
36 M	37 (1.1)

Notes. ^1^ Families described as ‘Other’ race include Asian American, Arab-American, and Native American families. ^2^ Family risks include being a teenaged mom, low levels of education, not in work or at school, single parent, and parent receiving welfare system.

**Table 2:** Descriptive Statistics and Correlations for the Study Variables.

	1.	2.	3.	4.	5.	6.	7.	8.	9.	10.	11.	12.	13.	14.
1. 14 M SUP	1.00						0.21	0.27 *	0.23 *	0.35 *	0.22 *	0.13		
2. 24 M SUP	0.52 *	1.00					0.17	0.23	0.17	0.32 *	0.16	0.12		
3. 36 M SUP	0.42 *	0.49 *	1.00				0.19	0.25 *	0.22 *	0.35 *	0.17	0.19		
4. 14 M EMR	0.16	0.10	0.12	1.00			0.15	0.18	0.15	0.15	0.11	0.08		
5. 24 M EMR	0.19	0.18	0.13	0.28 *	1.00		0.20 *	0.27 *	0.24 *	0.24 *	0.18	0.18		
6. 36 M EMR	0.18	0.13	0.19	0.17	0.37 *	1.00	0.25 *	0.38 *	0.33 *	0.31 *	0.26 *	0.31 *		
7. W-J Letter Word							1.00	0.53 *	0.38 *	0.45 *	0.34 *	0.29 *		
8. W-J Problem Solving								1.00	0.50 *	0.65 *	0.48 *	0.40 *		
9. Leiter Attention									1.00	0.47 *	0.37 *	0.35 *		
10. PPVT										1.00	0.48 *	0.45 *		
11. Book Knowledge											1.00	0.38 *		
12. Book Comprehension												1.00		
13. Program status (dummy)	0.05	0.04	0.13	−0.02	0.01	0.02	−0.04	−0.00	0.00	0.01	0.00	−0.01	1.00	
14. Family race (dummy)	−0.16	−0.13	−0.07	0.03	0.03	0.00	−0.03	−0.16	−0.06	−0.22	−0.05	−0.09	0.00	1.00
Mean	3.96	4.05	4.02	3.71	3.71	3.94	0.00	0.00	0.00	0.00	0.00	0.00		
Variance	1.09	1.18	0.87	0.49	0.63	0.60	0.99	0.99	0.99	0.99	0.99	0.99		
Minimum	1.00	1.00	1.67	1.00	1.00	1.33	−3.38	−4.57	−2.93	−3.35	−2.42	−2.84		
Maximum	7.00	7.00	6.33	5.00	5.00	5.00	3.40	2.19	2.60	3.90	1.43	1.07		
Sample size	2132	1513	789	2132	1513	789	1504	1505	1414	1483	1491	1485	2100	2100

Notes. * *p* ≤ 0.05; SUP = parent supportiveness; EMR = child emotion regulation; W-J is Woodcock Johnson; PPVT is Peabody Picture Vocabulary Test; and z-scores are indicators of cognitive school readiness construct.

**Table 3:** Results from the Regression of the Final Model Parameters on the Predictors.

	SUP (14 m)		EMR (14 m)		SUP (24 m)		EMR (24 m)		SUP (36 m)		EMR (36 m)		Cognitive School Readiness (Pre-K)	
	*b* (s.e.)	*p*	*b* (s.e.)	*p*	*b* (s.e.)	*p*	*b* (s.e.)	*p*	*b* (s.e.)	*p*	*b* (s.e.)	*p*	*b* (s.e.)	*p*
1. Race	−0.19 (0.03)	0.00	0.03 (0.02)	0.17	−0.08 (0.03)	0.01	0.03 (0.02)	0.15	0.01 (0.03)	0.87	0.01 (0.03)	0.69	−0.23 (0.04)	0.00
2. Program Status	0.10 (0.05)	0.03	−0.03 (0.03)	0.32	0.03 (0.05)	0.51	0.02 (0.04)	0.57	0.19 (0.06)	0.00	0.02 (0.05)	0.70	−0.12 (0.07)	0.07

Notes. SUP = Parent Supportiveness; EMR = Child Emotion Regulation; *b* = regression coefficient; s.e. = standard error of the coefficient; and *p* = statistical significance.

## Data Availability

The data presented in this study are not publicly available due to confidentiality agreement. The data can be requested from the Consortium Members of the Early Head Start Research and Evaluation program: https://doi.org/10.7910/DVN/GYQSSS.
